# Mitochondrial Heteroplasmy as a Marker for Premature Coronary Artery Disease: Analysis of the Poly-C Tract of the Control Region Sequence

**DOI:** 10.3390/jcm12062133

**Published:** 2023-03-08

**Authors:** Rebeca Lorca, Andrea Aparicio, Juan Gómez, Rut Álvarez-Velasco, Isaac Pascual, Pablo Avanzas, Francisco González-Urbistondo, Alberto Alen, Daniel Vázquez-Coto, Mar González-Fernández, Claudia García-Lago, Elías Cuesta-Llavona, César Morís, Eliecer Coto

**Affiliations:** 1 Área del Corazón, Hospital Universitario Central Asturias, 33011 Oviedo, Spain; 2 Instituto de Investigación Sanitaria del Principado de Asturias, ISPA, 33011 Oviedo, Spain; 3 Departamento de Morfología y Biología Celular, Universidad de Oviedo, 33003 Oviedo, Spain; 4 Unidad de Cardiopatías Familiares, Área del Corazón y Departamento de Genética Molecular, Hospital Universitario Central Asturias, 33011 Oviedo, Spain; 5 Redes de Investigación Cooperativa Orientadas a Resultados en Salud (RICORs), 28029 Madrid, Spain; 6 CIBER-Enfermedades Respiratorias, 28029 Madrid, Spain; 7 Laboratorio de Genética, Hospital Universitario Central Asturias, 33011 Oviedo, Spain; 8 Departamento de Medicina, Universidad de Oviedo, 33003 Oviedo, Spain; 9 CIBER-Enfermedades Cardiovasculares, 28029 Madrid, Spain

**Keywords:** atherosclerotic cardiovascular disease (ACVS), genetic testing, cardiovascular prevention, mitochondrial DNA (mtDNA)

## Abstract

Mitochondrial DNA (mtDNA) differs from the nuclear genome in many aspects: a maternal inheritance pattern; being more prone to acquire somatic de novo mutations, accumulative with age; and the possible coexistence of different mtDNA alleles (heteroplasmy). Mitochondria are key cellular organelles responsible for energy production and involved in complex mechanisms, including atherosclerosis. In this scenario, we aimed to evaluate mtDNA variants that could be associated with premature cardiovascular disease. We evaluated 188 consecutive patients presenting with premature myocardial infarction with ST elevation (STEMI) confirmed by coronary angiogram. mtDNA polymorphisms and clinical data were evaluated and compared with 271 individuals from the same population (control group). Tobacco consumption (80.85% vs. 21.21%, *p* < 0.01) and dyslipidemia (38.83% vs. 28.41%, *p* = 0.02) were significantly more frequent among STEMI patients. Moreover, C16223T mtDNA mutation and poly-C heteroplasmy were significantly more frequent among premature STEMI male patients than in controls. The OR associated C16223T mtDNA with the increased presence of cardiovascular risk factors. Our data suggest that mtDNA 16223T and heteroplasmy may be associated with unstable premature atherosclerosis disease in men. Moreover, the presence of cardiovascular risk factors (CVRFs) was associated with C16223T mtDNA, with a cumulative effect. Protective mitochondrial pathways are potential therapeutic targets. Preventing exposure to the damaging mechanisms associated with CVRFs is of utmost importance.

## 1. Introduction

Mitochondria contain their own genome, with structure, encoded genes, and other important differences from those of the nuclear genome [1]. Mitochondrial DNA (mtDNA) is a 16,569-bp double-stranded, circular DNA molecule that encodes for polypeptides forming the mitochondrial respiratory chain and mitochondrial tRNAs and rRNAs [1]. Most of the mitochondrial proteins are encoded by nuclear genes.

Germinal mutations in mtDNA can cause rare inherited diseases with a maternal inheritance pattern because only eggs contribute mitochondria to the zygote. On the other hand, mtDNA is more prone to acquire somatic mutations than nuclear DNA. In each cell cycle, the replicating mtDNA is at risk of de novo mutations that accumulate with age [2,3]. They may be generated by intrinsic cellular errors during DNA replication or repair or through exposure to mutagens, such as reactive oxygen species, environmental toxins, or lifestyle habits such as tobacco smoke or ultraviolet light exposure [3,4]. In addition, each cell has a variable number of mitochondria, and each mitochondrion contains a variable number of mtDNA molecules [1]. Consequently, mtDNA mutations do not follow the transmission pattern of the nuclear diploid genome. A particular nucleotide position in the same cell could be a single mtDNA genotype (homoplasmy), or there could coexist different mtDNA alleles (heteroplasmy) [2,5]. MtDNA mutations can contribute to human disease across a range of severity, from rare highly penetrant mutations causal for monogenic disorders that often affect the nervous system, muscles, heart, and endocrine organs, to variants that have a milder contribution to common complex traits and late-onset disorders. Many healthy humans harbor low levels (<1%) of mtDNA point mutations, either inherited or acquired. An increased burden of acquired mutations and heteroplasmy percentages may contribute to late-onset diseases. For instance, when the A3234G mtDNA mutation is present in a high percentage (>85%) often leads to MELAS disease. However, at lower heteroplasmy percentages (5–30%), it is associated with maternally inherited diabetes mellitus and deafness [6,7].

Mitochondrial heteroplasmy is particularly frequent in poly-cytosine tracts in the mtDNA control region. One of them contains the replication origin. These are prone to instability that results in different lengths of the poly-C tract and could affect the replication and stability of the mitochondrial genome. In this sense, an association of T16189C mtDNA polymorphism that increases the risk for poly-C instability has been associated with several diseases, including diabetes and coronary artery disease (CAD) [8,9].

Mitochondria are key cellular organelles responsible for energy production via cellular ATP generation by oxidative phosphorylation [10]. Furthermore, mitochondria are also involved in many essential cellular functions, including modulation of oxidation–reduction status, regulation of senescence and apoptosis, and also in immuno-mediated processes such as inflammation [3,11]. Mitochondrial dysfunction can directly promote cell death, inflammation, oxidative stress, and alter metabolism [12]. As a result, mitochondria are also involved in the complex mechanisms of atherosclerosis development, which includes low-density lipoprotein (LDL) oxidation, inflammation, cellular adhesion, fibrosis, and arterial wall calcification [10,13]. Some authors have studied the relationship between cardiovascular disease with mtDNA content and certain mtDNA mutations in peripheral blood lymphocytes [10]. It has been reported that certain mtDNA mutations and their increased heteroplasmy may lead to cell dysfunction due to a local increase in oxidative stress promoting atherosclerotic lesion formation [10]. However, many of these studies have been based on cohorts of subclinical atherosclerosis patients [14,15,16,17]. For instance, some homoplasmy mutations in mtDNA, such as C16223T, were more frequently detected in blood samples from asymptomatic atherosclerosis patients than in healthy persons [17].

In addition to rare pathogenic variants, the mitochondrial genome contains many common variants that originated in particular populations and spread with human migrations, defining the mitochondrial haplogroups. These haplogroups are transmitted from mother to offspring, and their frequencies vary between the different human populations. For instance, haplogroup H is the most frequent among Europeans but is almost absent among individuals of African or East Asian ascent. The study of haplogroups permitted to trace the migration of humans outside Africa and raised the concept of a mitochondrial Eve [18]. Mitochondrial haplogroups have been associated with differences in energy production and other physiological processes in which mitochondrial are involved. As a result, differences in the haplogroups have been associated with adaptation to exercise or susceptibility to developing several diseases [19,20,21,22]. In reference to CAD, some authors have reported a significantly increased risk for haplogroups H and T, but others failed to confirm these associations [8,23,24,25,26,27,28,29].

In this scenario, we aimed to evaluate whether common mtDNA variants that defined the European haplogroups could be associated with the risk of developing early-onset acute coronary artery disease (CAD). In addition, we sought to determine the degree of poly-C heteroplasmy in CAD. For this purpose, we compared a cohort of patients presenting with premature myocardial infarction with ST elevation (STEMI) with a control healthy group from the same population.

## 2. Materials and Methods

### 2.1. Study Population

In this study, we identified all consecutive patients referred to our center for emergency cardiac catheterization due to STEMI suspicion from 2018 to date. Our institution is both a reference center for primary angioplasty and a national reference center for inherited cardiac conditions.

Myocardial infarction (MI) is defined as an acute myocardial injury in the setting of evidence of acute myocardial ischemia [30,31]. Acute coronary syndrome (ACS) can be divided into ST-elevation myocardial infarction (STEMI), non-ST-elevation myocardial infarction (NSTEMI), and unstable angina. Patients with STEMI suspicion require emergency cardiac catheterization for primary angioplasty, as an acute total or subtotal coronary occlusion is suspected [30,31].

#### 2.1.1. Inclusion Criteria

- Consecutive patients referred to our center for emergency primary angioplasty from 2018 to date.
- STEMI definite diagnosis. MI type 1 is caused by atherothrombotic coronary artery disease (CAD) and is usually precipitated by atherosclerotic plaque. However, in type 2 MI, there is a demand–supply mismatch resulting in myocardial ischemia [30,31]. Atherothrombotic coronary artery disease (CAD) as the cause of the STEMI had to be confirmed by coronary angiogram [32].
- Patients with premature STEMI. Age limit to consider STEMI as “premature” was established according to DLCN criteria [33,34]. Only men 55 and women < 60 years old were included.
- Patients who accepted to participate in this study for investigational purposes.

#### 2.1.2. Exclusion Criteria

- Patients without significant coronary artery disease: individuals with major epicardial coronary arteries angiographically normal or with documented nonsignificant disease (<50% stenosis) by coronary angiogram [32].
- Patients with STEMI due to coronary artery dissection, as the etiopathogenesis differs from atherosclerosis background.
- Patients who died due to the ACS before being able to accept participation in the study.

### 2.2. Clinical Evaluation

We collected clinical data from this cohort. We reviewed their birth date, gender, age at STEMI, and classical cardiovascular risk factors (CVRF): high blood pressure (HBP), tobacco consumption, diabetes mellitus (DM), and dyslipidemia. Information about the familial history of premature CAD was also collected.

The research protocol followed institutional ethics guidelines. All patients with premature STEMI who wished to participate in the investigational project signed written consent to grant access to their genetic data for investigational purposes (CEImPA 2020.003).

### 2.3. Control Cohort

Control individual patients belong to a group of 271 individuals from the same population and belong to the RENASTUR cohort [35,36]. All of them were <65 years at the recruitment date, and 60% were male. They had no personal history of CAD. They were recruited with the only purpose of defining the frequency of the mtDNA variants in our population.

### 2.4. Genetic Testing

Patients and controls were genotyped for SIX mtDNA polymorphisms (G4580A, C7028T, A12308G, G13368A, G13708A, C16223T) that defined the most common European haplogroups (H, J, K, T, U, V, IWX), as previously reported [28,37]. The mtDNA 7028C is defined as haplogroup H. Those who had mtDNA 7028T were further genotyped for the mtDNA variants to determine the non-H haplogroups (J, KU, T, V, and WXI). The IWX haplogroups are characterized by mtDNA 16223T, in contrast to mtDNA 16223C, which characterizes the macro-haplogroup R that rooted the main European haplogroups.

**Figure 1:**
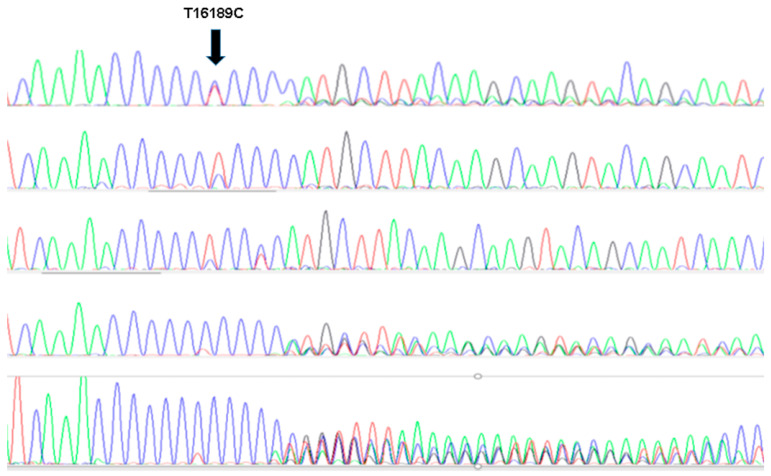
Mitochondrial DNA (mtDNA) sanger analysis. PCR fragments were sequenced to determine the degree of lenght heteroplasmy. The presence of a T interrupting the polyC was found in either homoplasmic or single nucleotide T/C heteroplasmics (three upper lines) while the absence of low T signal was found among individuals with complete lenght heteroplasmy.

We determined the 16,189 poly-C heteroplasmy of all the patients and controls. The mtDNA sequence between nucleotides 15,974 and 660 was amplified with primers 5′ CTCCACCATTAGCACCCAAAGCTAAG (forward) and 5′AGGACCAAACCTATTTGTTTATGGGGT (reverse). PCR fragments were Sanger sequenced with BigDye chemistry in ABI3130xl capillary equipment (Fisher Scientific). The degree of heteroplasmy in the 16,189 poly-C tracts was determined by the relative height of the fluorescent peaks (Figure 1).

### 2.5. Statistical Analysis

Statistical analyses were performed with SPSS v.19. Descriptive data for continuous variables are presented as mean ± SD and as frequencies or percentages for categorical variables. The Chi-square test or Fisher exact test was used to compare frequencies, whereas differences in continuous variables were evaluated with either Student’s *t*-test or Mann–Whitney U test. *p* < 0.05 was considered to be significant.

## 3. Results

A total of 188 patients presenting with premature STEMI were included in this study. The mean age was 49 years old ± 7.6 SD (48 ± 7.6 SD in men and 54.2 ± 5.2 SD in women). ACS was solely explained by a single vessel occlusion. However, additional significative CAD was found in nearly 40% of patients. The main clinical characteristics of the STEMI population compared with the control cohort are shown in Table 1.

Compared with control cases, both tobacco consumption and dyslipidemia were significantly more frequent among STEMI patients (Table 1). Mitochondrial analysis revealed that C16223T mtDNA mutation and poly-C heteroplasmy was significantly more frequent in premature STEMI cases than in controls. Haplogroup H was present in nearly half of the global cohort, and there were no differences between groups.

However, patients with premature STEMI were significantly more men, as expected by recruiting a cohort of patients with premature CAD. Therefore, we compared control-case individuals divided by gender. Not only tobacco consumption and dyslipidemia but also high blood pressure were significantly higher among male STEMI patients than in male control individuals.

We analyzed the mtDNA region encompassing the 16,184–16,193 poly-C tract that maps in the OriB origin of the replication sequence in the mitochondrial genome. This region is characterized by CT polymorphisms, and the presence of uninterrupted Cytosines is prone to instability visualized as sequence heteroplasmy (Figure 1). Heteroplasmy in this region was observed significantly more frequently in premature STEMI patients than in controls (Table 1 and Table 2). We found two types of heteroplasmy in this region. Dinucleotide 16,189 CT with no evidence for poly-C and 16,189 C with complete poly-C heteroplasmy (Figure 1). The first was found in 4 of the heteroplasmic controls and none of the patients who showed complete poly-C heteroplasmy with multiple poly-C sequences. This suggested a loss of the mtDNA replication-repair capacity among the patients that would increase the chance for heteroplasmy in regions prone to replication errors such as the poly-C tract.

In reference to the mtDNA variants, haplogroup H (tagged by 7028C) was slightly more common in the patients compared to non-H haplogroups (49% vs. 42%), a nonsignificant difference (*p* = 0.17). The ancestral 16223T allele that characterizes the IWX European macrohaplogroups was significantly more frequent in the patients. The other analyzed non-H haplogroups did not differ between the groups, although the low frequency of some of them and the reduced sample size underpowered the statistical differences (not represented in Table 1 and Table 2).

When unifying all five CVRF (male gender, previous/current smoker, high blood pressure, diabetes mellitus, dyslipidemia, and family history of premature CAD) in one variable, the OR associated with C16223T mtDNA increases to 1.68 (IC 1.2–2.35, *p* = 0.003). When analyzing this new variable at each interval, it can be seen that the association becomes stronger with the increase in CVRF (Table 3).

## 4. Discussion

Mitochondrial DNA molecules are frequently exposed to many damaging molecules called reactive oxygen species, considered the most important causes of mtDNA mutations. It is known that mtDNA damage and dysfunction may increase inflammation systems, and emerging evidence indicates that mtDNA damage can directly promote atherosclerosis [12]. Moreover, mitochondria are actively involved in lipid metabolism [12]. Reactive oxygen species (ROS) production can cause mtDNA damage and respiratory chain impairment, which further increases ROS formation and leads to oxidative stress, increased LDL oxidation, and, ultimately, the formation of atherogenic LDL species [12]. This makes mitochondria important players not only in initiation but also in the development of atherosclerosis and acute cardiovascular events. Therefore, identifying mtDNA mutations associated with atherosclerosis and preventing exposure to its damaging mechanisms is of utmost importance. As a result, point mtDNA mutations in blood cells associated with atherosclerosis are being actively studied, and the list of identified mutations is growing.

One of the strengths of this study is the sample selection, with restrictive inclusion criteria to identify only those patients presenting with a confirmed atherothrombotic acute event at a premature age. Selecting an adequate cohort of patients with a definite diagnosis is key to performing proper research for predisposing factors. For this purpose, we took advantage of the emergency primary angioplasty program from our center, registering all consecutive patients referred due to STEMI suspicion. Thanks to this selection based on coronary angiogram findings, only patients with confirmed MI type 1 due to atherothrombotic CAD were considered for further evaluation. Patients with type 2 MI, in which the etiopathogenesis of the myocardial ischemia is based on a demand–supply mismatch and not necessarily atherosclerosis underlying disease, could be excluded from this study. Moreover, in other to avoid other age-related confounded underlying factors, only patients with premature cardiovascular disease were included.

In this sense, results from other studies comparing genetics for predisposing factors based on heterogeneous cohorts, with vague definitions of atherosclerosis diseases and only discovered at a subclinical level [14,15,16,17], should be taken with caution. On the other hand, other important studies in human white blood cells comparing healthy controls with patients with significant CAD (properly evaluated by coronary angiogram) do not report any clinical information about the age of presentation or CAD presented as an acute coronary event [8,25,38,39,40]. Moreover, a Chinese study of mtDNA haplogroups found no association with the occurrence of CAD [24]. In this context, an interesting study performed on blood samples from healthy persons and patients with asymptomatic atherosclerosis identified seven homoplasmy mutations in mtDNA more frequently detected in atherosclerosis were identified, one of them C16223T [17].

Our study found that, compared with control cases, patients with premature STEMI included significantly more men with a history of current or previous tobacco consumption and dyslipidemia as the principal cardiovascular risk factors.

We did not find any significant difference in haplogroup H analysis. However, our finding revealed that 16223T mtDNA mutation was significantly more frequent in premature STEMI cases than in controls, and these significant differences remain significant when comparing men’s cases vs. controls. These results support the previous hypothesis that C16223T mtDNA mutation is associated with an increased risk of atherosclerosis [17]. Moreover, we found that the presence of CVRFs was associated with C16223T mtDNA, with an OR of 1.68 (IC 1.2–2.35, *p* = 0.003), and that the association became stronger with the increase in CVRF, as shown in Table 3. These findings support the idea that CVRFs such as tobacco may be the trigger of intrinsic cellular errors in mtDNA replication [3,4]. Allele 16223T was the ancestral compared to 16223C present in most of the European haplogroups. The exception is the rare European haplogroups IXW, all harboring 16223T. Interestingly, haplogroups with ancestral African alleles have been associated with increased oxidative stress and mtDNA damage [25].

We found a significantly increased frequency of poly-C heteroplasmy in STEMI patients. These results agreed with a previous study that reported an increased frequency of 16189C and poly-C tract heteroplasmy among coronary artery disease patients [8]. The associations of the 16,189 and other control region variants with cardiovascular diseases might be explained by the altered binding of proteins that regulate mtDNA replication [41,42,43]. Interestingly, the mitochondrial single-strand DNA-binding protein (mtSSB) would bind with a lower affinity to mtDNA with the 16189C variant, which was prone to poly-C instability [42]. Because the 16189C variant lies in the control region of mtDNA replication and transcription, it could be hypothesized that this variant might affect the mtDNA replication and the mtDNA content [44,45]. Thus, it is possible that the different mtSSB binding affinities for the 16,189 region change the rate of premature termination, thereby increasing the rate of mitochondrial genome loss and the speed of recovery following mtDNA damage [42].

A limitation of our study is that the heteroplasmy was not measured at different ages in the same individual, but only once. On the one hand, length heteroplasmy might increase in some individuals as a consequence of CAD risk factors, contributing to atherosclerosis progression. On the other hand, it could be the other way around. It is also possible that germline heteroplasmy was at a high degree in these individuals, increasing the risk of mitochondrial dysfunction and CAD. Because mtDNA heteroplasmy and instability have been reported in cardiac tissue from individuals with CAD [46], studies to determine whether the control region length variation differs between leukocytes and heart cells from these patients could be of special interest.

Finally, increased heteroplasmy has been associated with atherosclerotic disease by many postulated mechanisms, including chronic inflammation and possibly enhanced by tobacco consumption. Reduced mtDNA copy numbers were found in patients with histories of various adverse cardiovascular clinical events, and mtDNA copy number was inversely correlated with the extent of disease [47]. An increased risk of mtDNA instability and mtDNA content among carriers of the 16189C variant might explain these findings. The results presented in this study are consistent with this hypothesis. Heteroplasmy was also significantly more frequent in premature STEMI cases than in controls. We could conclude that the observed differences were characteristic of male patients and smokers. Larger cohorts, including more women presenting with premature STEMI, are needed to confirm these findings.

## 5. Conclusions

In our population, mitochondrial mtDNA 16223T and heteroplasmy wereassociated with unstable atherosclerosis disease, being more frequent in young male patients suffering from premature STEMI cases than in control individuals. Our findings also suggest that the presence of CVRFs was associated withC16223T mtDNA, with a cumulative effect. Protective mitochondrial pathways are potential therapeutic targets. Preventing exposure to the damaging mechanisms associated withCVRFs is of utmost importance.

## Figures and Tables

**Table 1:** Clinical and mitochondrial characteristics of patients presenting with premature ST-elevation myocardial infarction (premature STEMI = cases) and control cases.

	Total (*n* = 452)	Cases (*n* = 188)	Control (*n* = 271)	*p* *
Gender (men)	69.47% (314)	83.51% (157)	59.47% (157)	<0.0001
Cardiovascular risk factors				
Previous/current smoker	46.02% (208)	80.85% (152)	21.21% (56)	<0.0001
High blood pressure	23.67% (107)	27.13% (51)	21.21% (56)	0.1448
Diabetes mellitus	12.17% (55)	9.57% (18)	14.02% (37)	0.1546
Dyslipidemia	32.74% (148)	38.83% (73)	28.41% (75)	0.02
Family history of premature coronary artery disease		48 (25.5%)	unavailable	
Mitochondrial analysis				
16,189 Heteroplasmy	10.18% (46)	14.89% (28)	8% (22)	0.0051
16223T	7.08% (32)	13.30% (25)	4% (12)	<0.001
7028C Haplogroup H	45.13% (204)	48.94% (92)	42% (112)	0.1703

* Chi Square test.

**Table 2:** Clinical characteristics and mitochondrial characteristics divided by gender.

	Male			Female		
	Cases (*n* =157)	Controls (*n* = 157)	*p* *	Cases (*n* = 31)	Controls (*n* = 107)	*p* *
Cardiovascular Risk Factors						
Previous/current smoker	78.34% (123)	24.84% (39)	<0.001	93.55% (29)	15.89% (17)	<0.001
High blood pressure	28.03% (44)	16.56% (26)	0.015	22.58% (7)	28.04% (30)	0.546
Diabetes mellitus	9.55% (15)	11.46% (18)	0.581	9.68% (3)	17.76% (19)	0.405
Dyslipidemia	38.85% (61)	26.11% (41)	0.016	38.71% (12)	31.78% (34)	0.471
Mitochondrial Analysis						
16,189 Heteroplasmy	15.92% (25)	5.73% (9)	0.004	9.68% (3)	8.41% (9)	0.732
C16223T mtDNA	15.29% (24)	0% (0)	<0.001	3.23% (1)	6.54% (7)	0.487
7028CHaplogroup H	47.77% (75)	43.31% (68)	0.428	54.84% (17)	41.12% (44)	0.176

* Chi Square test.

**Table 3:** Association to C16223T mtDNA depending on the presence of classical cardiovascular risk factors.

	Odds Ratio	IC 95%	*p* Value
1 Factor	0.81	0.16–4.08	0.798
2 Factor	0.94	0.19–4.49	0.938
3 Factor	1.17	0.20–6.02	0.906
4 Factor	5.17	0.94–28.35	0.059
5 Factor	15.5	1.36–175.38	0.027

## Data Availability

The data that support the findings of this study are available from the corresponding author upon reasonable request. An Excel file with the raw data would be available for meta-analysis research.
